# Perityphlitis

**Published:** 1891-06

**Authors:** Joseph McEvoy

**Affiliations:** Atlanta, Ga.


### PERITYPHLITIS.

By JOSEPH McEVOY, M. D., Atlanta, Ga.

This disease, "although not at all uncommon," is frequently overlooked or misunderstood, notwithstanding the fact that a great deal has been said in our current medical literature about the affection and its proper treatment. What is known of the disease has not yet been embodied in our standard surgical works.

Since the plan of treating perityphlitic abscess by early incision was first adopted, the procedure has received considerable attention from American surgeons, and been fully endorsed by those who have published the results of their experience.

Further discussion of the subject is desirable in order that the disease may be readily recognized and submitted to proper treatment, and that we may be able to distinguish between those cases which tend to spontaneous recovery which are likely to terminate favorably without the aid of the knife, and those which necessarily call for surgical interference.

We therefore hope for a further discussion in order that we may gain additional information derived from the personal experience of some of our colleagues in regard to the pathology and treatment.

Various causes of the inflammatory condition under consideration have been assigned by systemic writers. The symptoms in nearly all cases point to a plastic inflammation of the connective tissue lying close to the cæcum. As the proximate cause of induration, lesion of the vermiform appendix is now understood as the immediate cause, the abscess resulting being primarily intraperitoneal.

Appendicitis is sometimes due to impaction of fæces in the cæcum—the presence of a foreign body in the organ. Idiopathic ulcer of the wall may lead to peritonitis and perforation, or the blood supply may be cut off from the organ by pressure of the surrounding viscera, thereby producing gangrene. The invariable occurrence of the disease on the right side is to be explained by reference to the cæcum and its appendix.

Perityphlitis does not always terminate in the formation of an abscess, as was once thought by some ; the disease often disappears by resolution, without any signs of pus.

The diagnosis, as a rule, is attended with considerable difficulty; the pain may be entirely absent until perforation has occurred.

It is usually present, however, and is very acute; is most marked in the region of the cæcum. This comes on suddenly; the pulse is accelerated, and the temperature is considerably raised; an indurated swelling may be detected either by palpation in the right iliac fossa or by digital exploration of the rectum. When found in the rectum it is a firm, elastic mass, tender to the touch, and situated near the region of the caput coli. When seen externally, it seems to be deep-seated, and is usually located on a line extending from the right superior iliac spine to the umbilicus and about two inches from the iliac spine.

The onset of the disease is usually severe, being characterized by nausea and vomiting. Sometimes the pain is diffused, but after the lapse of two or three days becomes localized; the temperature varies from 100° to 105° F.

Induration comes on usually within forty-eight hours after the commencement of the attack, and generally subsides soon after the abatement of the other symptoms when recovery takes place by resolution. So far as the early symptoms are concerned, it is impossible to say whether the case will end in resolution, or whether it will go on to suppuration.

Resolution rarely ever occurs before the eighth or ninth day; when it does occur it is usually accompanied by a reduction of the temperature and a perceptible diminution of the pain, distention and abdominal tenderness.

Sometimes the tumor subsides rapidly, at other times months elapse before it entirely disappears. If, however, the case should go on to suppuration, the symptoms, which have already been described, will increase in severity, together with rigor, sweating, tympanitis and an increased extent combined with a diminishing firmness of the tumor. These are the chief points which indicate the existence of pus; they usually appear about the end of the second week, and can be discriminated from those following resolution, inasmuch as they are more intense in character, the patient instead of getting better is continually growing worse; whereas when resolution has taken place, they are milder and the latter course of the disease is comparatively more favorable.

The treatment of perityphlitis in the commencement of the attack, should consist mainly of local depletion. Opium should be given in doses sufficient to allay the pain, but quantities sufficient to induce narcotism should never be given. Purging in the early part of the disease, as experience has taught, can do no harm, but acts beneficially by removing any accumulation of fæces which may be impacted in the intestines, which, if allowed to remain, only increases the suffering of the patient and is liable to aggravate the existing inflammation; it also aids in the diagnosis by excluding fæcal impaction in the cæcum, which might be the possible cause of the symptoms. We are told by some that here, as in peritonitis, purging should be withheld, but as the analogy between the two diseases is not so very close, and for the reasons above stated, I am of the opinion that purgation in the commencement of the affection is very essential, but should not be repeated, as a subsequent accumulation can be avoided by a careful restriction of the diet and by selection of light and nutritious articles of food, in connection with the treatment already briefly outlined. If absorption of the inflammatory products seem to be unduly retarded, blisters may be applied or mercurial applications may be resorted to.

If, however, the case should go on to suppuration and pus is evident, operative interference must not be postponed; an incision should be made, "running parallel with Poupart's ligament, about three inches in length directly over the tumor, cutting through the skin and subcutaneous fat; the deeper layers should then be divided until the *fascia transversalis* is reached; the wound should grow narrower as it increases in depth, in order that a subsequent hernial protrusion might be avoided. If, after having reached the *fascia transversalis* or abscess, fluctuation is evident, the abscess may be opened immediately, but if there is any doubt as to the locality of the abscess the *fascia* may be penetrated in various directions by means of a hypodermic needle, 'as Parker has recommended,' until the seat of the abscess is discovered, which should then be evacuated and drained. If the appendix be found diseased, it must be tied off by means of a ligature of silk close to the cæcum. A most perfect cleansing of the parts should be looked after, especially if perforation has occurred, taking care to use all antiseptic precautions. The wound should not be sewn up, but should be packed with iodiformized gauze, which should be removed after firm adhesions have been established."

